# Assessment of the Thyroid Functions among Female Flight Attendants

**DOI:** 10.3390/ijerph18041929

**Published:** 2021-02-17

**Authors:** Małgorzata Radowicka, Anna Madej, Bronisława Pietrzak, Mirosław Wielgoś

**Affiliations:** Department of Obstetrics and Gynecology, Medical University of Warsaw, 02-015 Warsaw, Poland; anna.madejj@yahoo.com (A.M.); bronislawa.pietrzak@wum.edu.pl (B.P.); miroslaw.wielgos@wum.edu.pl (M.W.)

**Keywords:** flight attendants, shift work, thyroid function, thyroid-stimulating hormone

## Abstract

Introduction: Epidemiological observations indicate that stewardesses are exposed to reproductive and endocrine system disturbances. The aim of the study was to assess of thyroid function disturbances as well as to identify factors affecting the thyroid function among stewardesses working both within one time zone and on long-distance flights. Material and methods: The cross-sectional study covered 103 women aged 23–46. The study group (I) was divided into two subgroups: group Ia comprising stewardesses flying within one flight zone and group Ib stewardesses working on long-distance flights. The control group (II) were women of reproductive age who sought medical assistance due to marital infertility in whom the male factor was found to be responsible for problems with conception in the course of the diagnostic process. The assessment included: age, BMI, menstrual cycle regularity, length of work, frequency of flying, thyroid stimulating hormone (TSH) concentration, level of free thyroxine (fT4), antibodies to thyroglobulin (aTG) and to thyroperoxidase (aTPO), prolactin concentration, sex hormone binding globulin (SHGB) concentration, total cholesterol, and low density lipoprotein (LDL) fraction. Descriptive methods and inferential statistics methods were used to compile the data. Results: The difference between the concentrations of TSH in the study group (2.59 mcIU/mL) and the control group (1.52 mcIU/mL) was statistically significant (*p* < 0.01). An elevated titer of thyroid antibodies (aTPO and/or aTG) was revealed in 46.3% of stewardesses and in 15.1% of patients from the control group (*p* < 0.001). Groups Ia and Ib in individual concentrations were not statistically significant. The frequency of occurrence of an elevated titer of thyroid antibodies depended on the length of work in the study group (*p* > 0.05). No statistically significant difference was found in patients spending up to 60 h a month flying and in patients spending more than 60 h flying, the percentage of the occurrence of thyroid antibodies was 50% and 43.5, respectively. Conclusions: The occurrence in stewardesses of a higher TSH concentration than in the control group can signify that stewardesses are burdened with a higher risk of the development of hypothyroidism in the future. The character of the work of stewardesses (frequency of flying as well as length of work) does not affect the immunological profile of the thyroid.

## 1. Introduction

Shift work has an adverse impact on health [1]. In accordance with the Polish Labor Code (Art. 151), the night shift is defined as the time covering 8 h between 21:00 and 07:00 while a worker whose work schedule involves every day at least 3 h of work during the night hours or whose at least ¼ of the working time in the settlement period falls to night shift hours is a night shift worker [2]. According to the Main Statistical Office, in Poland, night shift work is performed by 417,700 women, including flight attendants [3]. Epidemiological studies reveal that shift work can cause, among others, diseases of the cardiovascular system, sleep disturbances, and breast cancer in women [4,5]. The negative influence of night shift work on health is linked mainly to the disturbance of the circadian rhythm disrupting, among others, the secretion of prolactin, cortisol, or the growth hormone [6,7]. The level of the thyroid stimulating hormone (TSH) changes with the circadian rhythm and the sleep pattern [8]. The TSH concentration is one of the most sensitive methods of the evaluation of the thyroid function [9]. Studies evaluating the relation between thyroid diseases and night shift work are very few.

Research shows that stress connected with the performance of the profession contributes to the development of autoimmune diseases, among others, such as systemic lupus erythematosus (SLE), rheumatoid arthritis, or Graves–Basedow disease [10,11]. In the case of flight attendants, the character of work involves, in addition, exposure to prolonged stress. Studies clearly shows that more stressful part of flight is landing, especially just before touchdown [12]. The action of prolonged stress can make these women belong to the risk group for the development of autoimmune diseases.

The aim of the study was to determine the frequency of occurrence of thyroid function disturbances as well as to assess the influence of the frequency of flying and the length of work on the development of this disturbance among flight attendants working both within one time zone (more take-offs and more landings) and on long-distance flights.

## 2. Material and Methods

### 2.1. Study Design

The cross-sectional study covered 103 Polish women aged 23–46. The women were qualified in the Department of Obstetrics and Gynecology, Medical University of Warsaw and examined in the Department of Gynecological Endocrinology, Medical University of Warsaw, Poland in the years 2013–2016. Approval for this study was obtained from the Medical University of Warsaw, Ethics Board (KB/254/2013, 12 November 2013). Notification about the study to be conducted was also given to the trade unions of LOT Polish Airlines and EUROLOT Polish Airlines. Exclusion criteria included use of hormonal drugs (including contraceptives) up to 6 months prior to the study, use of drugs inducing the activity of hepatic enzymes which can affect the hormonal economy of the organism, history of chronic renal insufficiency and liver cirrhosis, and diagnosis of menopause in women over 40, according to the WHO criteria. 

The trade unions gather over five hundred flight attendants. Seventy-four flight attendants responded to the letter of invitation, forty-three of them qualified for the study. All patients gave their written informed consent for study participation. The study group (I) consisted of female flight attendants flying within one time zone as well as those serving long-distance flights. Qualified for the study were female flight attendants who consented to take part in the study, were of reproductive age, and worked under the night shift system. The control group (II) were 60 women of reproductive age who sought medical assistance due to marital infertility in whom the male factor was found to be responsible for problems with conception in the course of the diagnostic process. Those women work full time (160 h per month) in the public sector as clerks. They do not do night shift work and do not report being exposed to excessive work-related stress. 

### 2.2. Sample Collection and Analysis

**Figure 1:**
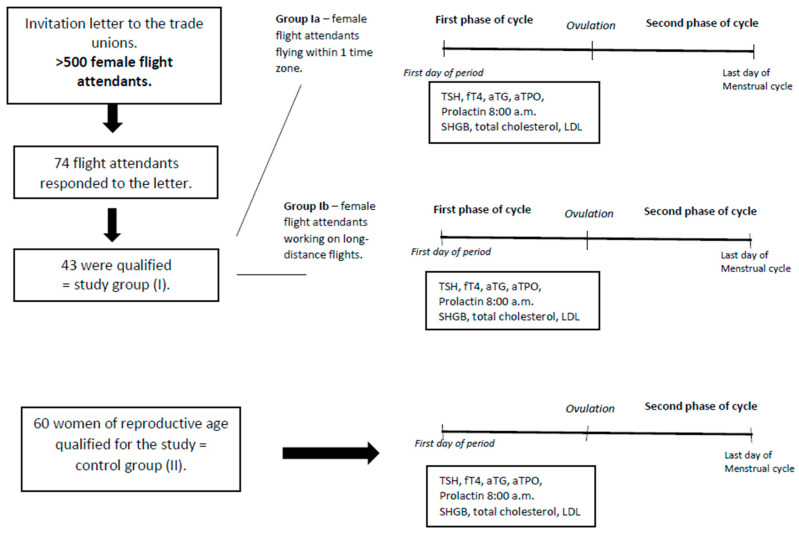
Study design. TSH—thyroid-stimulating hormone; fT4—free thyroxine; aTG—antibodies to thyroglobulin; aTPO—antibodies to thyroperoxidase; SHGB—sex hormone binding globulin; LDL—low-density lipoprotein.

The examination was performed in accordance with the protocol for diagnosis of hormonal disorders at the Department of Gynecological Endocrinology, Medical University of Warsaw. The blood tests were carried out between the 4th–6th day of the cycle (1st phase of the cycle). The patients were not examined directly after night shifts. Every woman came for the examination after a good night’s sleep. The study group was divided into two subgroups: Group Ia (*n* = 17) comprising female flight attendants flying within one time zone and Group Ib (*n* = 26) female flight attendants working on long-distance flights. The assessment included age, BMI, and menstrual cycle regularity. The assessment did not include smoking, alcohol consumption, physical activity, or dietary habits. The study design is presented in Figure 1. Menstrual cycles of 25–35 days were adopted as regular. The frequency of flying was expressed in terms of the number of flying hours a month. Assessing the influence of the frequency of flying on the development of hormonal disturbances, the study group of the female flight attendants was divided into two subgroups: women working less than 60 h a month and women working 60 or more hours a month. Assessing the influence of the length of work on the development of hormonal disturbances the study group of the female flight attendants was divided into two subgroups: women working less than 15 years and women working 15 or more years.

The thyroid function was determined through the evaluation of the TSH (normal range: 0.35–4.94 mcIU/mL), the level of free thyroxine—fT4 (normal range: 9.01–19.05 pmol/L), the level of antibodies to thyroglobulin—aTG (normal range: <4.11 IU/mL) and thyroperoxidase—aTPO (normal range: <5.61 IU/mL). Assessing the potential consequences of thyroid dysfunction, the prolactin concentration (normal range: 5–35 ng/mL), sex hormone binding globulin (SHBG) concentration (normal range: 19.8–155.2 nmol/L), total cholesterol (normal range: 115–190 mg/dL) and low density lipoprotein (LDL) fraction (normal range: >115 mg/dL) was measured at 8 a.m. in 1st phase of the cycle. The TSH and prolactin concentration and level of antibodies in a blood sample collected from the antecubital vein was assessed with an automated enzyme-immunoassay and with ELISA method.

### 2.3. Statistical Analysis

Descriptive methods and inferential statistics methods were used to compile the data. The randomness of the study sample was examined in terms of the age and length of work of the patients. To this end, a series of tests was applied checking the hypothesis that the way in which patients were selected could be deemed random. Knowing the order in which the patients registered for the study, the randomness of the sample in terms of age and length of work was confirmed. For the qualitative features the following characteristics were calculated: arithmetic mean (x), median (Me), standard deviation (SD), and coefficient of variation (v%). The chi-square independence test was applied to compare the frequency of the occurrence of individual varieties of features in the study groups and in the subgroups. Where the expected numbers were lower than 5, Yates correction was used in the calculation of the value of the chi-square test. Prior to the comparison of mean values in the study groups and subgroups, the conformity of the distributions of the analyzed measurable variables with the normal distribution was checked with the help of the Shapiro–Wilk test. As the distributions of the majority of the analyzed variables differed significantly from the normal distribution, the comparison of the mean values was chosen to be made with the help of the non-parametric test, rather than the parametric test. As the samples were independent, the Mann–Whitney test was used to compare the mean values. To study the correlation between the measurable variables, the rank correlation coefficient was applied due to distributions significantly different from the normal distribution. The differences between mean values (or frequencies) were found statistically significant when the calculated value of a relevant test was equal or higher than the critical value from relevant tables, with an adequate number of the degrees of freedom and the probability of error *p* < 0.05.

## 3. Results

Table 1 shows the characteristics of the study group and the control group, including the subgroups of women flying within one time zone and women serving long-distance flights. 

The mean age of the respondents in the control group (II) was 34.0, SD 4.09 years. Women from the study group (I) were aged 25–43 and the average age was 34.7, SD 4.41 years. 

The length of work in the study group ranged from 6 to 25 years. On average, women worked 14.3 SD 5.06 years. Half of the women studied worked 15 or more years. The women from the study group spent 47 to 85 h a month flying, on average 64.6 SD 7.89 h, while half of them spent 65 or more hours a month flying.

**Figure 2:**
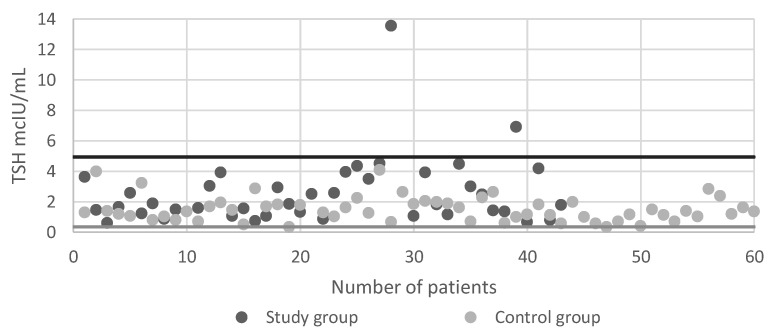
The TSH concentration in the study group and in the control group.

**Figure 3:**
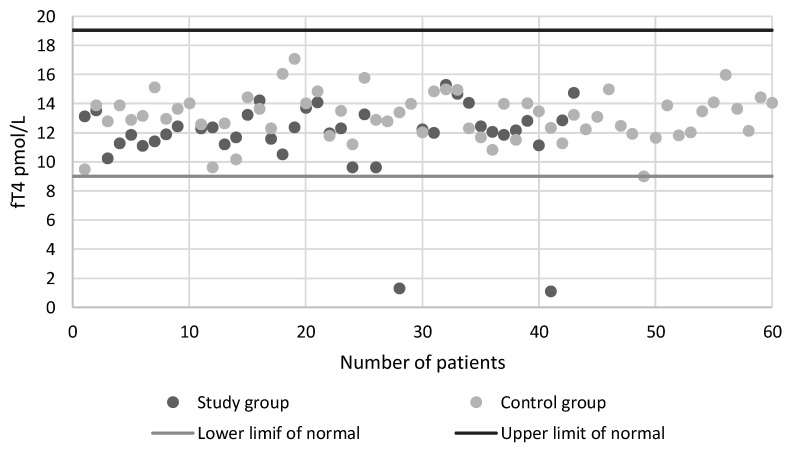
The fT4 level in the study group and in the control group.

Table 2 shows the concentration of TSH and level of fT4 in the groups studied. The difference between the concentrations of TSH in the study group and the control group was statistically significant (*p* < 0.01). A statistically significant difference was also revealed between Group Ib and the control group (2.75 mcIU/mL vs. 1.52 mcIU/mL). The difference between the level of Ft4 in the study group and the control group was statistically significant (*p* < 0.01). A significant difference was also found between Group Ia and the control group (11.55 pmol/L vs. 13.13 pmol/L) as well as between Group Ib and the control group (12.01 pmol/L vs. 13.13 pmol/L). Though study group and Group Ib have increased TSH level and decreased fT4 level when compared to control group, levels were in normal range. The charts of TSH level and fT4 level is presented in Figure 2 and Figure 3.

The high TSH concentration and the low fT4 concentration, allowing to diagnose hypothyroidism, was present in two people in the study group (4.7%) while in the control group no such cases were found.

An elevated titer of thyroid antibodies (aTPO and/or aTG) was revealed in 19 flight attendants and in 8 patients from the control group (46.3% vs. 15.1%). The difference was statistically significant (*p* < 0.001). No statistically significant difference was found in the occurrence of an elevated titer of aTPO and/or aTG between Groups Ia and Ib, but it turned out that these antibodies are found much more frequently in Group of Ib than in Group Ia (52% vs. 37.5%). Correlation was examined between the mean concentration of thyroid antibodies in the study group and the frequency of its occurrence in hyperprolactinemia. No statistically significant correlation was found. An elevated titer of aTPO and/or aTG was slightly more common in patients with hyperprolactinemia than in patients with normal prolactin concentration: 47.4% and 42.9%. The frequency of occurrence of an elevated titer of thyroid antibodies depended on the length of work in the study group. No statistically significant correlation was found. Elevated titers of thyroid antibodies were present with an approximate frequency among flight attendants working longer than 15 years and among the flight attendants working less than 15 years: 45% vs. 47.6%. The correlation between the frequency of the occurrence of elevated titer of aTPO and/or aTG and the number of flight hours a month of the flight attendants was studied. No statistically significant difference was found between patients spending up to 60 h a month flying and patients spending more than 60 h flying; the percentage of the occurrence of elevated titer of thyroid antibodies was 50% and 43.5, respectively.

Table 3 shows the SHBG concentration, total cholesterol, and LDL fraction in the study group and the control group. No statistically significant difference was found between the groups studied (I, II, Ia, Ib).

## 4. Discussion

Hypothyroidism is one of the most common endocrine diseases. It is defined as growth in the TSH concentration and decrease in the fT4 concentration. In the general population, the disease is reported in 0.2–5.3% of people, 8–9 times more frequently in women [13,14]. In the group of patients covered by our study, the occurrence of hypothyroidism was not higher than in the general population. It was found only in air hostesses working on long-distance flights and amounted to 4.7%.

The study confirmed the presence of a significantly higher mean TSH concentration in flight attendants than in the control group (2.59 vs. 1.52). Studies assessing the correlation between shift work and the TSH level are very few. A similar correlation was found by So-Hyun Noon and others. They examined the TSH level in 967 women working in a hospital in Korea, among which 546 did night shifts and 421 only day shifts. They found that the mean TSH concentration in the women working night shifts was 0.303 mlU/L higher, this being a significant difference [15]. Due to character of the profession performed, female flight attendants have several potential factors could cause increased TSH concentration and decreased fT4 concentration. TSH levels exhibit a circadian rhythm with peak concentration at 2–4 a.m. and trough concentration at 4–8 p.m. [16]. Some studies suggest that sleep deprivation promotes fluctuations in the TSH circadian rhythm, which increases the likelihood of the higher TSH levels [17]. Moreover, disturbance of the circadian rhythm in women induced changes in the menstrual cycle by modulating the female hormones [18]. Epidemiological evidence suggests a role of estrogen in the pathogenesis of thyroid disease [19]. Furthermore, night work is associated with nocturnal eating, which may affect hormonal levels of TSH, insulin, and glucagon [20], so it is possible that such eating habits could lead to increased TSH level.

Subclinical hypothyroidism is characterized by a normal fT4 concentration and an elevated TSH level. In the general population, subclinical hypothyroidism is reported in 4–15% of people [21]. The presence of autoantibodies against thyroid peroxidase causes there to be a 25–50% risk of subclinical hypothyroidism becoming overt hypothyroidism within 20 years [22]. In the group of flight attendants studied, elevated titer of antibodies (aTPO and aTG) was obtained in as much as 46.3% of the patients, the majority of them being women working on long-distance flights. Our study findings did not reveal a significant influence of the length of work or frequency of flying on the occurrence of an elevated level of antibodies. The significantly higher TSH concentration in flight attendants and the elevated titer of antibodies in nearly a half of the patients from the study group can suggest that flight attendants belong to the high-risk group as regards the development of hypothyroidism.

Stress induces autoimmune disturbances by modulating the immune response. Research has shown that shift work-related stress contributes to intensifying hyperthyroidism in the course of Graves–Basedow disease. Works describing correlations between autoimmune hypothyroidism and shift work are very few. Magrini and others assessed the level of TPO and the concentration of TSH in 220 shift workers and 422 day-workers. They based the diagnosis of autoimmune subclinical hypothyroidism on high aTPO levels and high TSH concentrations. The thus-defined disease was diagnosed in 8% of people doing shift work and 4% of people working only day shifts. Increased levels of TPO antibodies were found in 14% of shift workers against 9% of people working day-shifts [23]. The fact that our study revealed the presence of an elevated level of TPO and/or TG antibodies in almost every second patient from the study group seems to suggest that it can be due to shift work-related stress as well as the very character of the flight attendant’s profession.

## 5. Conclusions

1. The occurrence in flight attendants of a higher TSH concentration than in the control group can signify that flight attendants are burdened with a higher risk of the development of hypothyroidism in the future.
2. The character of the work of flight attendants (frequency of flying as well as length of work) does not affect the immunological profile of the thyroid.
3. The study findings suggest that the frequent appearance of the elevated aTPO and aTG levels can be linked to stress and shift work, but further studies are needed to confirm these results.

## Figures and Tables

**Table 1:** The characteristics of the study group and control group, including the subgroups of women flying within one time zone and women serving long-distance flights. Time of work in air was significantly different between groups Ia and Ib.

Variable	Control Group (II) (*N* = 60)			Study Group (I) (*N* = 43)			*p*	Group Ia (*N* = 17)			Group Ib (*N* = 26)			*p*
	Min	Max	M ± SD	Min	Max	M ± SD		Min	Max	M ± SD	Min	Max	M ± SD	
**Age (years)**	23	46	34.0 ± 4.09	25	43	34.7 ± 4.41	0.203	26	43	34.1 ± 4.26	25	40	35.2 ± 4.53	0.219
**BMI (kg/m^2^)**	17.5	37	22.7 ± 3.78	17.5	29.0	22.4 ± 2.71	0.995	17.5	28	21.7 ± 2.67	19	29	22.8 ± 2.71	0.28
**Seniority** **(years)**				6	25	14.3 ± 5.06		6	18	13.0 ± 4.23	6	25	15.2 ± 5.44	0.084
**Time of work in air (h/month)**				47	85	64.6 ± 7.89		47	70	59.5 ± 6.80	55	85	67.9 ± 6.81	**0.001**

Bolded is *p* < 0.001. Control group (II)—60 women of reproductive age who sought medical assistance due to marital infertility in whom the male factor was found to be responsible for problems with conception in the course of the diagnostic process; Study group (I)—43 female flight attendants; Group Ia—17 female flight attendants flying within one time zone; Group Ib—26 female flight attendants working on long-distance flights.

**Table 2:** The concentration of TSH and level of fT4 in the groups studied. Despite the significant differences, the values of TSH and free T4 were in normal range.

	Control Group (II) (*N* = 60)			Study Group (I) (*N* = 43)			*p*	Group Ia (*N* = 17)			Group Ib (*N* = 26)			*p*
	Min	Max	M ± SD	Min	Max	M ± SD		Min	Max	M ± SD	Min	Max	M ± SD	
**TSH (mIU/L)**	0.35	4.10	1.52 ± 0.83	0.62	13.56	2.59 ± 2.25	**0.0037**	0.62	4.36	2.35 ± 1.40	0.69	13.56	2.75 ± 2.67	0.915
**fT4 (pmol/L)**	9.0	17.08	13.13 ± 1.62	1.12	15.32	11.83 ± 2.75	**0.0044**	1.12	14.76	11.55 ± 3.15	1.33	15.32	12.01 ± 2.51	0.989

Bolded is *p* < 0.05. Control group (II)—60 women of reproductive age who sought medical assistance due to marital infertility in whom the male factor was found to be responsible for problems with conception in the course of the diagnostic process; Study group (I)—43 female flight attendants; Group Ia—17 female flight attendants flying within one time zone; Group Ib—26 female flight attendants working on long-distance flights.

**Table 3:** The concentrations of the prolactin, SHBG, total cholesterol, and LDL fraction in individual groups.

	Control Group (II) (*N* = 60)			Study Group (I) (*N* = 43)			*p*	Group Ia (*N* = 17)			Group Ib (*N* = 26)			*p*
	Min	Max	M ± SD	Min	Max	M±SD		Min	Max	M±SD	Min	Max	M±SD	
**Prolactin (ng/mL) 8:00 am**	5.56	123.9	22.6 ± 16.1	14.82	63.24	35.4 ± 11.8	**<0.001**	15.97	56.76	33.7 ± 12.9	14.82	63.24	36.3 ± 11.2	0.425
**SHBG (nmol/L)**	24.6	175.7	72 ± 36.1	38.1	164.9	71.35 ± 25.8	0.491	38.1	122.2	65.87 ± 23.7	41.3	164.9	74.64 ± 27	0.154
**Total cholesterol (mg/dL)**	105	244	171.3 ± 30.3	132	240	176.3 ± 23.5	0.397	140	210	169.5 ± 20.4	132	240	180.5 ± 24.7	0.133
**LDL fraction (mg/dL)**	48	173	98 ± 29.9	52	168	98.7 ± 23.4	0.683	68	142	94.1 ± 18.8	52	168	101.7 ± 26	0.238

Bolded is *p* < 0.001. Control group (II)—60 women of reproductive age who sought medical assistance due to marital infertility in whom the male factor was found to be responsible for problems with conception in the course of the diagnostic process; Study group (I)—43 female flight attendants; Group Ia—17 female flight attendants flying within one time zone; Group Ib—26 female flight attendants working on long-distance flights.

## Data Availability

The data presented in this study are available on request from corresponding author. The data are not publicly available due to polish law regulation does not allow for public access of medical data.

## References

[1] Nena E., Katsaouni M., Steiropoulos P., Theodorou E., Constantinidis T.C., Tripsianis G. (2018). Effect of shift work on sleep, health, and quality of life of health-care workers. Indian J. Occup. Environ. Med..

[2] The Polish Parliament (1974). Act of 5 July 1974 Labour Code. J. Laws.

[3] Rozkrut D., Rozkrut D. (2019). Employment. Yearbook of Labour Statistics.

[4] Wang X.-S., Armstrong M.E.G., Cairns B.J., Key T.J., Travis R.C. (2011). Shift work and chronic disease: The epidemiological evidence. Occup. Med..

[5] Szkiela M., Kusideł E., Makowiec-Dąbrowska T., Kaleta D. (2020). Night shift work—A Risk factor for breast cancer. Int. J. Environ. Res. Public Health.

[6] Weibel L., Brandenberger G. (1998). Disturbances in hormonal profiles of night workers during their usual sleep and work times. J. Biol. Rhythm..

[7] Morris C.J., Aeschbach D., Scheer F.A. (2012). Circadian system, sleep and endocrinology. Mol. Cell. Endocrinol..

[8] Kalsbeek A., Fliers E. (2013). Daily regulation of hormone profiles. Handb. Exp. Pharmacol..

[9] Soldin O.P., Chung S.H., Colie C. (2013). The use of TSH in determining thyroid disease: How does it impact the practice of medicine in pregnancy?. J. Thyroid. Res..

[10] Stojanovich L., Marisavljevich D. (2008). Stress as a trigger of autoimmune disease. Autoimmun. Rev..

[11] Falgarone G., Heshmati H.M., Cohen R., Reach G. (2013). Role of emotional stress in the pathophysiology of Graves’ disease. Eur. J. Endocrinol..

[12] Roscoe A.H. (1978). Stress and workload in pilots. Aviat. Space Environ. Med..

[13] Chaker L., Bianco A.C., Jonklaas J., Peeters R.P. (2017). Hypothyroidism. Lancet.

[14] Chiovato L., Magri F., Carlé A. (2019). Hypothyroidism in context: Where we’ve been an where we’re going. Adv. Ther..

[15] So-Hyun M., Bum-Joon L., Seong-Jin K., Hwan-Cheol K. (2016). Relationship between thyroid stimulating hormone and night shift work. Ann. Occup. Environ. Med..

[16] Patel Y.C., Alford F.P., Burger H.G. (1972). The 24-hour plasma thyrotrophin profile. Clin. Sci..

[17] Parker D.C., Rossman L.G., Pekary A.E., Hershman J.M. (1987). Effect of 64-hour sleep deprivation on the circadian waveform of thyrotropin (TSH): Further evidence of sleep-related inhibition of TSH release. J. Clin. Endocrinol. Metab..

[18] Davis S., Mirick D.K., Chen C., Stanczyk F.Z. (2012). Night shift work and hormone levels in women. Cancer Epidemiol. Biomark. Prev..

[19] Manole D., Schildknecht B., Gosnell B., Adams E., Derwahl M. (2001). Estrogen promotes growth of human thyroid tumor cells by different molecular mechanisms. J. Clin. Endocrinol. Metab..

[20] Holmbäck U., Forslund A., Lowden A., Forslund J., Akerstedt T., Lennernäs M., Hambraeus L., Stridsberg M. (2003). Endocrine responses to nocturnal eating-possible implications for night work. Eur. J. Nutr..

[21] Sviridonova M.A., Fadeyev V.V., Sych Y.P., Melnichenko G.A. (2013). Clinical significance of TSH circadian variability in patients with hypothyroidism. Endocr. Res..

[22] Hennessy J.V., Espaillat R. (2015). Diagnosis and management of subclinical hypothyroidism in elderly adults: A review of the literature. J. Am. Geriatr. Soc..

[23] Magrini A., Pietroiusti A., Coppeta L., Babbucci A., Barnaba E., Papadia C., Iannaccone U., Boscolo P., Bergamaschi E., Bergamaschi A. (2006). Shift work and autoimmune thyroid disorders. Int. J. Immunopathol. Pharmacol..

